# Yttrium ethyl­enediammonium squarate tetra­hydrate

**DOI:** 10.1107/S1600536811011251

**Published:** 2011-04-07

**Authors:** Louiza Zenkhri, Nathalie Audebrand, Thierry Bataille

**Affiliations:** a Faculté des Sciences et Technologie et Sciences de la Matière, Université Kasdi Merbah Ouargla, Route Gardaia, Ourgla, Algeria; b Laboratoire Sciences Chimiques de Rennes (CNRS, UMR 6226), Université de Rennes 1, Avenue du Général Leclerc, 35042 Rennes Cedex, France

## Abstract

The title compound, {(C_2_H_10_N_2_)_1.5_[Y(C_4_O_4_)_3_(H_2_O)_4_]}_*n*_ {system­atic name: *catena*-poly[sesqui(ethyl­enediammonium) [[tetra­aquabis­(squarato-κ*O*)yttrium(III)]-μ-squarato-κ^2^ *O*:*O*′]]}, was synthesized by slow evaporation of an acid solution. The asymetric unit contains one yttrium cation in an anti­prismatic environnement, three squarate groups, one and a half protonated ethyl­enediamine mol­ecules and four water mol­ecules. YO_8_  polyhedra are connected through bis­(mono­dentate) squarates, leading to infinite zigzag chains, in between which are located ammonium groups. A framework of hydrogen bonds between protonated amine N atoms, water mol­ecules and squarate anions ensures the cohesion of the structure.

## Related literature

For a related structure, see: Kazerouni *et al.* (1994) ▶. The title compound was obtained together with two polymorphs of (C_2_H_10_N_2_)(HC_4_O_4_)_2_(H_2_O) (Mathew *et al.*, 2002 ▶; Zenkhri *et al.*, 2011) ▶. For related yttrium squarates with potassium, see: Mahé & Bataille (2004 ▶).
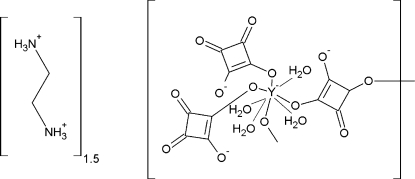


## Experimental

### 

#### Crystal data

(C_2_H_10_N_2_)_1.5_[Y(C_4_O_4_)_3_(H_2_O)_4_]
*M* *_r_* = 590.27
Monoclinic, 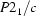

*a* = 8.9780 (2) Å
*b* = 13.2864 (3) Å
*c* = 18.3970 (4) Å
β = 90.935 (1)°
*V* = 2194.20 (8) Å^3^
*Z* = 4
Mo *K*α radiation
μ = 2.75 mm^−1^
*T* = 293 K
0.22 × 0.14 × 0.12 mm

#### Data collection

Nonius KappaCCD diffractometer
Absorption correction: analytical (de Meulenaer & Tompa, 1965) ▶ *T*_min_ = 0.583, *T*_max_ = 0.734
23285 measured reflections
5016 independent reflections
4140 reflections with *I* > 2σ(*I*)
*R*_int_ = 0.065

#### Refinement

*R*[*F* ^2^ > 2σ(*F* ^2^)] = 0.041
*wR*(*F* ^2^) = 0.103
*S* = 1.10
5016 reflections
316 parameters
H-atom parameters constrained
Δρ_max_ = 1.77 e Å^−3^
Δρ_min_ = −0.62 e Å^−3^

### 

Data collection: *COLLECT* (Nonius, 2000 ▶); cell refinement: *DIRAX/LSQ* (Duisenberg, 1992 ▶); data reduction: *EVALCCD* (Duisenberg *et al.*, 2003 ▶); program(s) used to solve structure: *SIR97* (Altomare *et al.*, 1999 ▶); program(s) used to refine structure: *SHELXL97* (Sheldrick, 2008 ▶); molecular graphics: *DIAMOND* (Brandenburg & Berndt, 2001 ▶); software used to prepare material for publication: *WinGX* publication routines (Farrugia, 1999 ▶).

## Supplementary Material

Crystal structure: contains datablocks I, global. DOI: 10.1107/S1600536811011251/ru2003sup1.cif

Structure factors: contains datablocks I. DOI: 10.1107/S1600536811011251/ru2003Isup2.hkl

Additional supplementary materials:  crystallographic information; 3D view; checkCIF report

## Figures and Tables

**Table 1:** Hydrogen-bond geometry (Å, °)

*D*—H⋯*A*	*D*—H	H⋯*A*	*D*⋯*A*	*D*—H⋯*A*
N1—H12⋯O3^i^	0.87	2.21	3.027 (4)	157
N1—H12⋯O9^ii^	0.87	2.36	2.929 (4)	123
N1—H11⋯O4^i^	0.87	2.36	2.995 (4)	131
N1—H11⋯O7^ii^	0.87	2.28	2.987 (4)	138
N1—H13⋯O6^ii^	0.87	2.36	2.970 (4)	127
N1—H13⋯O12^ii^	0.87	2.26	3.032 (4)	148
N2—H23⋯O2	0.85	1.87	2.707 (4)	167
N2—H21⋯O4^i^	0.96	1.92	2.832 (4)	159
N2—H21⋯O7^ii^	0.96	2.46	2.952 (4)	112
N2—H22⋯O5^iii^	0.95	2.01	2.929 (4)	163
N3—H31⋯O4	0.87	2.22	2.843 (4)	129
N3—H32⋯O8^iv^	0.87	2.18	2.788 (4)	127
N3—H33⋯O*W*1^v^	0.87	2.40	2.997 (4)	126
O*W*1—H911⋯O11^v^	0.94	1.74	2.676 (3)	175
O*W*1—H912⋯O5	0.93	1.78	2.687 (3)	166
O*W*2—H922⋯O11	0.94	1.95	2.885 (4)	173
O*W*2—H921⋯O10^v^	0.94	1.79	2.726 (3)	179
O*W*3—H931⋯O8^vi^	0.93	1.94	2.857 (4)	172
O*W*3—H932⋯O1^i^	0.94	2.04	2.954 (5)	163
O*W*4—H942⋯O5^vi^	0.92	1.86	2.779 (3)	172
O*W*4—H941⋯O10^vii^	0.93	1.86	2.761 (4)	163

Symmetry codes: (i) 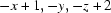; (ii) 
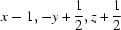
; (iii) 
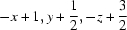
; (iv) 
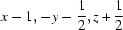
; (v) 
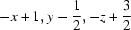
; (vi) 
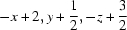
; (vii) 
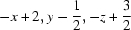
.

**Fig. 1.**
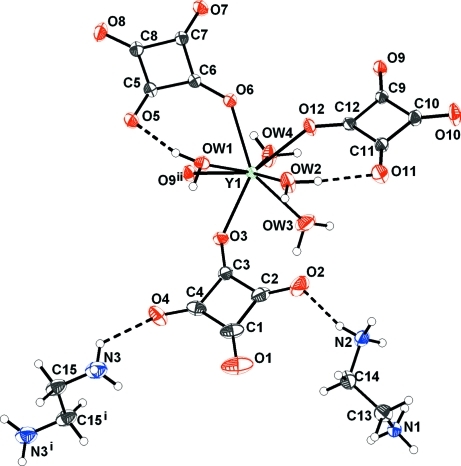
View of the molecular structure of the title compound. Ellipsoids are shown at the 50% probability level. (i) 1 - x, -1 - y, 2 - z; (ii) 2 - x, -1/2 + y, 3/2 - z.

**Fig. 2.**
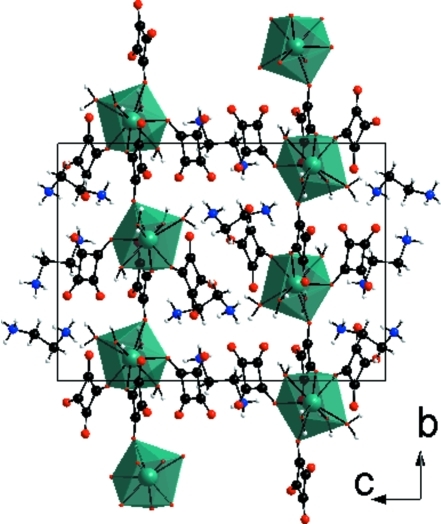
View of the structure of the title compound along the a axis displaying chains alternating with amine groups.

## supplementary crystallographic information

### Comment

In the course of a study on mixed squarate of amines and metals, the role of the amine group has been investigated in the topology of the organic-inorganic framework. The synthesis led to a new mixed squarate of yttrium and ethylenediamine.

Yttrium is eightfold coordinated in the shape of a square antiprism. YO_8_ polyhedra are connected along the *b* axis through bis(monodentate) squarates in the form of zigzag chains (Figures 1, 2). Amine groups are located between the chains (Figure 2) and are connected to them through hydrogen bonds involving oxygen atoms from squarate groups (Table 1). Other hydrogen bonds between water molecules and squarate groups contribute also to the formation of a three-dimensional molecular framework (Table 1). One ethylenediammonium possesses a *gauche* conformation as already reported for this molecule (Kazerouni *et al.*, 1994).

### Experimental

The title compound, Y(C_4_O_4_)_3_(C_2_H_10_N_2_)_1.5_4H_2_O was prepared from an aquous solution (20 ml) of dissolved yttrium nitrate (0.5 mmol), ethylenediamine (0.1 mmol) and 3,4-dihydroxy-3-cyclobutene-1,2-dione, also named squaric acid (0.1 mmol). The slow evaporation at room temperature leads after some hours to the formation of the title compound together with two polymorphs of (HC_4_O_4_)_2_(C_2_H_10_N_2_)(H_2_O) (Mathew *et al.*, 2002; Zenkhri *et al.*., 2011).

### Refinement

All H atoms were found from Fourier difference maps. H atoms attached to C were fixed geometrically and treated as riding with C—H = 0.97 Å with *U*_iso_(H) = 1.2*U*_eq_. As H atoms attached to N and O are not geometrically tightened, they were refined using restraints of N–H = 0.89 (1)Å and O–H = 0.97 (1)Å with *U*_iso_(H) = 1.2*U*_eq_(N) and *U*_iso_(H) = 1.5*U*_eq_(O), respectively. In the last cycles of refinement, they were treated as riding on their parent atoms.

### Crystal data

**Table:**

(C_2_H_10_N_2_)_1.5_[Y(C_4_O_4_)_3_(H_2_O)_4_]	*F*(000) = 1204
*M**_r_* = 590.27	*D*_x_ = 1.787 Mg m^−^^3^
Monoclinic, *P*2_1_/*c*	Mo *K*α radiation, λ = 0.71073 Å
*a* = 8.9780 (2) Å	Cell parameters from 57148 reflections
*b* = 13.2864 (3) Å	θ = 2.9–27.5°
*c* = 18.3970 (4) Å	µ = 2.75 mm^−^^1^
β = 90.935 (1)°	*T* = 293 K
*V* = 2194.20 (8) Å^3^	Prism, colourless
*Z* = 4	0.22 × 0.14 × 0.12 mm

### Data collection

**Table:**

Nonius KappaCCD diffractometer	5016 independent reflections
Radiation source: fine-focus sealed tube	4140 reflections with *I* > 2σ(*I*)
horizonally mounted graphite crystal	*R*_int_ = 0.065
Detector resolution: 9 pixels mm^-1^	θ_max_ = 27.5°, θ_min_ = 2.9°
CCD scans	*h* = −11→11
Absorption correction: analytical (de Meulenaer & Tompa, 1965)	*k* = −17→17
*T*_min_ = 0.583, *T*_max_ = 0.734	*l* = −23→23
23285 measured reflections	

### Refinement

**Table:**

Refinement on *F*^2^	Primary atom site location: structure-invariant direct methods
Least-squares matrix: full	Secondary atom site location: difference Fourier map
*R*[*F*^2^ > 2σ(*F*^2^)] = 0.041	Hydrogen site location: inferred from neighbouring sites
*wR*(*F*^2^) = 0.103	H-atom parameters constrained
*S* = 1.10	*w* = 1/[σ^2^(*F*_o_^2^) + (0.0368*P*)^2^ + 4.9093*P*] where *P* = (*F*_o_^2^ + 2*F*_c_^2^)/3
5016 reflections	(Δ/σ)_max_ = 0.002
316 parameters	Δρ_max_ = 1.77 e Å^−^^3^
0 restraints	Δρ_min_ = −0.62 e Å^−^^3^

### Special details

**Table:**

Geometry. All e.s.d.'s (except the e.s.d. in the dihedral angle between two l.s. planes) are estimated using the full covariance matrix. The cell e.s.d.'s are taken into account individually in the estimation of e.s.d.'s in distances, angles and torsion angles; correlations between e.s.d.'s in cell parameters are only used when they are defined by crystal symmetry. An approximate (isotropic) treatment of cell e.s.d.'s is used for estimating e.s.d.'s involving l.s. planes.
Refinement. Refinement of *F*^2^ against ALL reflections. The weighted *R*-factor *wR* and goodness of fit *S* are based on *F*^2^, conventional *R*-factors *R* are based on *F*, with *F* set to zero for negative *F*^2^. The threshold expression of *F*^2^ > 2σ(*F*^2^) is used only for calculating *R*-factors(gt) *etc*. and is not relevant to the choice of reflections for refinement. *R*-factors based on *F*^2^ are statistically about twice as large as those based on *F*, and *R*- factors based on ALL data will be even larger.

### Fractional atomic coordinates and isotropic or equivalent isotropic displacement parameters (Å^2^)

**Table:**

	*x*	*y*	*z*	*U*_iso_*/*U*_eq_	
Y1	0.81967 (3)	0.09385 (2)	0.775395 (16)	0.01739 (9)	
OW1	0.6982 (2)	−0.02399 (18)	0.69447 (13)	0.0255 (5)	
H911	0.6156	−0.0626	0.7085	0.038*	
H912	0.7647	−0.0720	0.6785	0.038*	
OW2	0.5760 (3)	0.15385 (18)	0.74497 (16)	0.0332 (6)	
H921	0.4774	0.1303	0.7446	0.050*	
H922	0.5568	0.2216	0.7550	0.050*	
OW3	0.7609 (4)	0.1825 (2)	0.88343 (19)	0.0653 (12)	
H931	0.7880	0.2376	0.9116	0.098*	
H932	0.7345	0.1447	0.9243	0.098*	
OW4	1.0544 (3)	0.16009 (19)	0.82212 (15)	0.0332 (6)	
H941	1.1451	0.1438	0.8013	0.050*	
H942	1.0623	0.2267	0.8356	0.050*	
C1	0.3965 (4)	−0.0751 (3)	0.9370 (2)	0.0344 (9)	
O1	0.2824 (3)	−0.0919 (3)	0.97182 (19)	0.0618 (10)	
C2	0.4625 (4)	0.0166 (3)	0.90558 (19)	0.0308 (8)	
O2	0.4298 (4)	0.1078 (2)	0.90299 (18)	0.0567 (9)	
C3	0.5884 (4)	−0.0427 (2)	0.88126 (17)	0.0215 (6)	
O3	0.7096 (2)	−0.02378 (17)	0.84855 (13)	0.0260 (5)	
C4	0.5259 (4)	−0.1337 (3)	0.91110 (19)	0.0270 (7)	
O4	0.5696 (3)	−0.2235 (2)	0.91517 (16)	0.0389 (6)	
C5	0.9863 (4)	−0.0763 (2)	0.60978 (17)	0.0212 (6)	
O5	0.8971 (3)	−0.14412 (17)	0.62986 (14)	0.0283 (5)	
C6	1.0043 (4)	0.0306 (2)	0.62387 (17)	0.0212 (6)	
O6	0.9443 (3)	0.09575 (17)	0.66401 (13)	0.0278 (5)	
C7	1.1316 (4)	0.0349 (2)	0.57414 (18)	0.0228 (6)	
O7	1.2148 (3)	0.10214 (18)	0.55251 (16)	0.0356 (6)	
C8	1.1158 (4)	−0.0751 (2)	0.56315 (18)	0.0227 (7)	
O8	1.1858 (3)	−0.14202 (19)	0.53033 (16)	0.0368 (6)	
C9	0.8814 (3)	0.4399 (2)	0.72880 (17)	0.0190 (6)	
O9	1.0131 (2)	0.46219 (16)	0.71244 (13)	0.0244 (5)	
C10	0.7446 (4)	0.4939 (2)	0.74815 (18)	0.0225 (6)	
O10	0.7095 (3)	0.58392 (18)	0.75490 (17)	0.0360 (6)	
C11	0.6683 (3)	0.3960 (2)	0.75669 (18)	0.0216 (6)	
O11	0.5417 (3)	0.3661 (2)	0.77318 (16)	0.0351 (6)	
C12	0.8066 (3)	0.3451 (2)	0.73838 (17)	0.0203 (6)	
O12	0.8488 (3)	0.25554 (17)	0.73267 (15)	0.0316 (6)	
N1	0.1150 (3)	0.2173 (2)	1.13530 (16)	0.0276 (6)	
H11	0.1863	0.2579	1.1230	0.033*	
H12	0.1531	0.1615	1.1520	0.033*	
H13	0.0618	0.2454	1.1688	0.033*	
C13	0.0190 (4)	0.1952 (3)	1.0706 (2)	0.0315 (8)	
H131	−0.0192	0.2581	1.0511	0.038*	
H132	−0.0654	0.1553	1.0858	0.038*	
C14	0.0985 (4)	0.1398 (3)	1.0114 (2)	0.0317 (8)	
H141	0.1488	0.0817	1.0323	0.038*	
H142	0.0257	0.1156	0.9760	0.038*	
N2	0.2096 (3)	0.2041 (2)	0.97398 (16)	0.0300 (7)	
H21	0.2778	0.2278	1.0107	0.036*	
H22	0.1607	0.2561	0.9477	0.036*	
H23	0.2683	0.1717	0.9464	0.036*	
N3	0.4125 (4)	−0.4070 (3)	0.93482 (18)	0.0392 (8)	
H31	0.4774	−0.3779	0.9072	0.047*	
H32	0.3940	−0.3685	0.9718	0.047*	
H33	0.3306	−0.4175	0.9100	0.047*	
C15	0.4730 (4)	−0.5043 (3)	0.9610 (2)	0.0348 (9)	
H151	0.5550	−0.5246	0.9306	0.042*	
H152	0.3963	−0.5555	0.9574	0.042*	

### Atomic displacement parameters (Å^2^)

**Table:**

	*U*^11^	*U*^22^	*U*^33^	*U*^12^	*U*^13^	*U*^23^
Y1	0.01584 (14)	0.01229 (13)	0.02423 (15)	0.00006 (11)	0.00636 (10)	0.00037 (12)
OW1	0.0212 (11)	0.0241 (12)	0.0313 (12)	−0.0036 (9)	0.0061 (9)	−0.0030 (10)
OW2	0.0161 (11)	0.0211 (12)	0.0625 (18)	0.0014 (9)	0.0049 (11)	0.0036 (12)
OW3	0.099 (3)	0.0388 (17)	0.060 (2)	−0.0325 (18)	0.055 (2)	−0.0255 (15)
OW4	0.0302 (13)	0.0235 (12)	0.0456 (16)	−0.0003 (10)	−0.0048 (11)	−0.0100 (11)
C1	0.0197 (16)	0.058 (3)	0.0259 (18)	−0.0034 (16)	0.0021 (13)	0.0039 (16)
O1	0.0269 (15)	0.104 (3)	0.055 (2)	−0.0107 (17)	0.0177 (14)	0.011 (2)
C2	0.0284 (18)	0.042 (2)	0.0228 (17)	0.0099 (16)	0.0066 (14)	0.0032 (15)
O2	0.068 (2)	0.0505 (19)	0.0524 (19)	0.0358 (17)	0.0266 (17)	0.0089 (15)
C3	0.0219 (15)	0.0236 (16)	0.0190 (15)	0.0011 (13)	0.0032 (12)	0.0002 (12)
O3	0.0234 (11)	0.0234 (12)	0.0317 (13)	0.0022 (9)	0.0116 (10)	0.0058 (10)
C4	0.0217 (16)	0.0335 (18)	0.0257 (17)	−0.0081 (14)	−0.0004 (13)	0.0042 (14)
O4	0.0408 (15)	0.0259 (13)	0.0498 (17)	−0.0086 (12)	0.0008 (13)	0.0090 (12)
C5	0.0258 (16)	0.0155 (14)	0.0224 (15)	0.0002 (12)	0.0017 (12)	0.0011 (11)
O5	0.0297 (12)	0.0183 (11)	0.0373 (14)	−0.0060 (10)	0.0110 (10)	−0.0002 (10)
C6	0.0249 (15)	0.0158 (14)	0.0232 (16)	−0.0003 (12)	0.0055 (12)	0.0006 (12)
O6	0.0352 (13)	0.0170 (11)	0.0317 (13)	−0.0019 (10)	0.0166 (10)	−0.0012 (10)
C7	0.0238 (16)	0.0182 (15)	0.0265 (16)	0.0022 (12)	0.0068 (13)	0.0024 (12)
O7	0.0364 (14)	0.0196 (12)	0.0517 (16)	−0.0027 (11)	0.0238 (12)	0.0033 (11)
C8	0.0243 (16)	0.0182 (15)	0.0258 (16)	0.0005 (12)	0.0025 (13)	−0.0006 (12)
O8	0.0382 (14)	0.0227 (13)	0.0500 (17)	0.0037 (11)	0.0193 (12)	−0.0081 (11)
C9	0.0183 (14)	0.0160 (14)	0.0229 (15)	0.0008 (11)	0.0034 (12)	−0.0004 (12)
O9	0.0177 (11)	0.0168 (11)	0.0390 (14)	−0.0030 (9)	0.0086 (9)	−0.0019 (10)
C10	0.0195 (15)	0.0193 (15)	0.0289 (17)	0.0032 (12)	0.0023 (12)	−0.0001 (13)
O10	0.0253 (12)	0.0161 (12)	0.0668 (19)	0.0064 (10)	0.0064 (12)	−0.0027 (12)
C11	0.0174 (14)	0.0193 (15)	0.0282 (16)	0.0020 (12)	0.0045 (12)	0.0017 (13)
O11	0.0181 (11)	0.0275 (13)	0.0602 (18)	0.0025 (10)	0.0131 (11)	0.0071 (12)
C12	0.0185 (14)	0.0177 (14)	0.0247 (16)	0.0015 (12)	0.0040 (12)	0.0036 (12)
O12	0.0275 (12)	0.0132 (11)	0.0546 (16)	0.0009 (9)	0.0149 (11)	0.0046 (11)
N1	0.0320 (15)	0.0198 (13)	0.0314 (16)	0.0027 (12)	0.0110 (12)	0.0010 (12)
C13	0.0216 (16)	0.0338 (19)	0.039 (2)	0.0014 (14)	0.0029 (14)	0.0051 (16)
C14	0.040 (2)	0.0239 (17)	0.0309 (19)	0.0005 (15)	−0.0044 (15)	0.0000 (14)
N2	0.0353 (16)	0.0283 (15)	0.0267 (15)	0.0109 (13)	0.0061 (12)	−0.0009 (12)
N3	0.0367 (18)	0.046 (2)	0.0351 (17)	−0.0108 (16)	0.0085 (14)	−0.0034 (15)
C15	0.0296 (18)	0.044 (2)	0.031 (2)	−0.0111 (16)	0.0079 (15)	−0.0092 (16)

### Geometric parameters (Å, °)

**Table:**

Y1—O3	2.297 (2)	C8—O8	1.250 (4)
Y1—O12	2.304 (2)	C9—O9	1.261 (4)
Y1—O9^i^	2.314 (2)	C9—C12	1.439 (4)
Y1—O6	2.351 (2)	C9—C10	1.471 (4)
Y1—OW3	2.377 (3)	O9—Y1^ii^	2.314 (2)
Y1—OW2	2.386 (2)	C10—O10	1.244 (4)
Y1—OW1	2.409 (2)	C10—C11	1.479 (4)
Y1—OW4	2.428 (2)	C11—O11	1.247 (4)
OW1—H911	0.9405	C11—C12	1.458 (4)
OW1—H912	0.9255	C12—O12	1.254 (4)
OW2—H921	0.9388	N1—C13	1.487 (5)
OW2—H922	0.9354	N1—H11	0.8700
OW3—H931	0.9277	N1—H12	0.8700
OW3—H932	0.9382	N1—H13	0.8700
OW4—H941	0.9307	C13—C14	1.504 (5)
OW4—H942	0.9214	C13—H131	0.9700
C1—O1	1.237 (4)	C13—H132	0.9700
C1—C2	1.476 (5)	C14—N2	1.491 (5)
C1—C4	1.484 (5)	C14—H141	0.9700
C2—O2	1.249 (5)	C14—H142	0.9700
C2—C3	1.454 (5)	N2—H21	0.9571
C3—O3	1.277 (4)	N2—H22	0.9479
C3—C4	1.445 (5)	N2—H23	0.8528
C4—O4	1.257 (5)	N3—C15	1.479 (5)
C5—O5	1.265 (4)	N3—H31	0.8700
C5—C6	1.453 (4)	N3—H32	0.8700
C5—C8	1.456 (4)	N3—H33	0.8700
C6—O6	1.264 (4)	C15—C15^iii^	1.512 (8)
C6—C7	1.476 (4)	C15—H151	0.9700
C7—O7	1.234 (4)	C15—H152	0.9700
C7—C8	1.482 (4)		
			
O3—Y1—O12	152.48 (8)	C5—C6—C7	90.7 (3)
O3—Y1—O9^i^	73.35 (8)	C6—O6—Y1	135.7 (2)
O12—Y1—O9^i^	131.37 (8)	O7—C7—C6	134.9 (3)
O3—Y1—O6	137.19 (8)	O7—C7—C8	136.6 (3)
O12—Y1—O6	68.55 (8)	C6—C7—C8	88.4 (2)
O9^i^—Y1—O6	77.00 (8)	O8—C8—C5	133.6 (3)
O3—Y1—OW3	75.14 (9)	O8—C8—C7	136.0 (3)
O12—Y1—OW3	81.46 (10)	C5—C8—C7	90.3 (2)
O9^i^—Y1—OW3	116.50 (12)	O9—C9—C12	132.6 (3)
O6—Y1—OW3	147.03 (9)	O9—C9—C10	137.1 (3)
O3—Y1—OW2	87.89 (8)	C12—C9—C10	90.2 (2)
O12—Y1—OW2	73.57 (8)	C9—O9—Y1^ii^	139.9 (2)
O9^i^—Y1—OW2	150.02 (8)	O10—C10—C9	135.0 (3)
O6—Y1—OW2	103.86 (9)	O10—C10—C11	135.7 (3)
OW3—Y1—OW2	79.37 (13)	C9—C10—C11	89.2 (2)
O3—Y1—OW1	74.04 (8)	O11—C11—C12	133.8 (3)
O12—Y1—OW1	116.61 (9)	O11—C11—C10	137.0 (3)
O9^i^—Y1—OW1	81.66 (8)	C12—C11—C10	89.2 (2)
O6—Y1—OW1	71.68 (8)	O12—C12—C9	132.7 (3)
OW3—Y1—OW1	137.23 (10)	O12—C12—C11	135.9 (3)
OW2—Y1—OW1	70.61 (9)	C9—C12—C11	91.4 (2)
O3—Y1—OW4	114.74 (9)	C12—O12—Y1	145.1 (2)
O12—Y1—OW4	71.35 (9)	C13—N1—H11	109.5
O9^i^—Y1—OW4	71.44 (8)	C13—N1—H12	109.5
O6—Y1—OW4	83.21 (9)	H11—N1—H12	109.5
OW3—Y1—OW4	74.20 (11)	C13—N1—H13	109.5
OW2—Y1—OW4	138.51 (9)	H11—N1—H13	109.5
OW1—Y1—OW4	146.71 (8)	H12—N1—H13	109.5
Y1—OW1—H911	122.4	N1—C13—C14	113.6 (3)
Y1—OW1—H912	110.8	N1—C13—H131	108.8
H911—OW1—H912	103.1	C14—C13—H131	108.8
Y1—OW2—H921	138.7	N1—C13—H132	108.8
Y1—OW2—H922	116.5	C14—C13—H132	108.8
H921—OW2—H922	98.3	H131—C13—H132	107.7
Y1—OW3—H931	142.9	N2—C14—C13	112.5 (3)
Y1—OW3—H932	117.9	N2—C14—H141	109.1
H931—OW3—H932	92.4	C13—C14—H141	109.1
Y1—OW4—H941	122.0	N2—C14—H142	109.1
Y1—OW4—H942	120.4	C13—C14—H142	109.1
H941—OW4—H942	105.7	H141—C14—H142	107.8
O1—C1—C2	133.9 (4)	C14—N2—H21	106.7
O1—C1—C4	136.9 (4)	C14—N2—H22	110.2
C2—C1—C4	89.2 (3)	H21—N2—H22	113.9
O2—C2—C3	134.3 (4)	C14—N2—H23	114.2
O2—C2—C1	136.1 (3)	H21—N2—H23	101.0
C3—C2—C1	89.6 (3)	H22—N2—H23	110.5
O3—C3—C4	133.1 (3)	C15—N3—H31	109.5
O3—C3—C2	135.3 (3)	C15—N3—H32	109.5
C4—C3—C2	91.6 (3)	H31—N3—H32	109.5
C3—O3—Y1	142.0 (2)	C15—N3—H33	109.5
O4—C4—C3	133.9 (3)	H31—N3—H33	109.5
O4—C4—C1	136.4 (3)	H32—N3—H33	109.5
C3—C4—C1	89.6 (3)	N3—C15—C15^iii^	110.7 (4)
O5—C5—C6	135.6 (3)	N3—C15—H151	109.5
O5—C5—C8	134.0 (3)	C15^iii^—C15—H151	109.5
C6—C5—C8	90.4 (2)	N3—C15—H152	109.5
O6—C6—C5	136.6 (3)	C15^iii^—C15—H152	109.5
O6—C6—C7	132.6 (3)	H151—C15—H152	108.1

Symmetry codes: (i) −*x*+2, *y*−1/2, −*z*+3/2; (ii) −*x*+2, *y*+1/2, −*z*+3/2; (iii) −*x*+1, −*y*−1, −*z*+2.

### Hydrogen-bond geometry (Å, °)

**Table:**

*D*—H···*A*	*D*—H	H···*A*	*D*···*A*	*D*—H···*A*
N1—H12···O3^iv^	0.87	2.21	3.027 (4)	157
N1—H12···O9^v^	0.87	2.36	2.929 (4)	123
N1—H11···O4^iv^	0.87	2.36	2.995 (4)	131
N1—H11···O7^v^	0.87	2.28	2.987 (4)	138
N1—H13···O6^v^	0.87	2.36	2.970 (4)	127
N1—H13···O12^v^	0.87	2.26	3.032 (4)	148
N2—H23···O2	0.85	1.87	2.707 (4)	167
N2—H21···O4^iv^	0.96	1.92	2.832 (4)	159
N2—H21···O7^v^	0.96	2.46	2.952 (4)	112
N2—H22···O5^vi^	0.95	2.01	2.929 (4)	163
N3—H31···O4	0.87	2.22	2.843 (4)	129
N3—H32···O8^vii^	0.87	2.18	2.788 (4)	127
N3—H33···OW1^viii^	0.87	2.40	2.997 (4)	126
OW1—H911···O11^viii^	0.94	1.74	2.676 (3)	175
OW1—H912···O5	0.93	1.78	2.687 (3)	166
OW2—H922···O11	0.94	1.95	2.885 (4)	173
OW2—H921···O10^viii^	0.94	1.79	2.726 (3)	179
OW3—H931···O8^ii^	0.93	1.94	2.857 (4)	172
OW3—H932···O1^iv^	0.94	2.04	2.954 (5)	163
OW4—H942···O5^ii^	0.92	1.86	2.779 (3)	172
OW4—H941···O10^i^	0.93	1.86	2.761 (4)	163

Symmetry codes: (iv) −*x*+1, −*y*, −*z*+2; (v) *x*−1, −*y*+1/2, *z*+1/2; (vi) −*x*+1, *y*+1/2, −*z*+3/2; (vii) *x*−1, −*y*−1/2, *z*+1/2; (viii) −*x*+1, *y*−1/2, −*z*+3/2; (ii) −*x*+2, *y*+1/2, −*z*+3/2; (i) −*x*+2, *y*−1/2, −*z*+3/2.
